# Risk Factors of Microalbuminuria among Patients with Type 2 Diabetes Mellitus in Korea: A Cross-Sectional Study Based on 2019–2020 Korea National Health and Nutrition Examination Survey Data

**DOI:** 10.3390/ijerph20054169

**Published:** 2023-02-25

**Authors:** Eun Sook Bae, Jung Yi Hur, Hyung Soon Jang, Jeong Suk Kim, Hye Seung Kang

**Affiliations:** 1 Good Aein Medical Care Hospital, Busan 47889, Republic of Korea; 2 Department of Nursing, Saekyung University, Yeongwol-gun 26239, Gangwon-do, Republic of Korea

**Keywords:** diabetes mellitus, diabetic nephropathy, microalbuminuria, risk factor

## Abstract

Diabetes mellitus is a chronic disease with high economic and social burdens. This study aimed to determine the risk factors of microalbuminuria among patients with type 2 diabetes mellitus. Microalbuminuria is predictive of early-stage renal complications and subsequent progression to renal dysfunction. We collected data on type 2 diabetes patients who participated in the 2019–2020 Korea National Health and Nutrition Examination Survey. The risk factors for microalbuminuria among patients with type 2 diabetes were analyzed using logistic regression. As a result, the odds ratios were 1.036 (95% confidence interval (CI) = 1.019–1.053, *p* < 0.001) for systolic blood pressure, 0.966 (95% CI = 0.941–0.989, *p* = 0.007) for high-density lipoprotein cholesterol level, 1.008 (95% CI = 1.002–1.014, *p =* 0.015) for fasting blood sugar level, and 0.855 (95% CI = 0.729–0.998, *p* = 0.043) for hemoglobin level. A significant strength of this study is the identification of low hemoglobin level (i.e., anemia) as a risk factor for microalbuminuria in patients with type 2 diabetes. This finding implies that the early detection and management of microalbuminuria can prevent the development of diabetic nephropathy.

## 1. Introduction

Microalbuminuria is defined as the persistent elevation of albumin excretion (30–300 mg/day) in urine. This range is higher than that of normoalbuminuria (<30 mg/day) but lower than that of albuminuria (<300 mg/day) [1]. Microalbuminuria is a known early predictor of kidney and cardiovascular diseases as well as diabetes and hypertension [2,3,4,5], and an increased albumin concentration in the urine is a result of kidney disease [6]. The kidney is mainly comprised of microvessels, and diabetic nephropathy occurs due to defects in the glomerular filtration barrier caused by damage in the renal microvasculature of the glomerulus; the lack of moderate regulation of blood pressure or blood glucose levels in patients with hypertension or diabetes, respectively, could accelerate this condition [7]. Diabetic nephropathy is initially characterized by a drop in the glomerular filtration rate, followed by a period of microalbuminuria. In a considerable number of diabetic patients, the proportion of those with microalbuminuria increases by 20% each year; then, they are eventually diagnosed with diabetic nephropathy when albuminuria is detected [8,9]. Approximately 20%~40% of diabetic patients develop diabetic nephropathy; it is a major complication of diabetes that reduces the patient’s quality of life. Hence, the early diagnosis of diabetic nephropathy is critical to enable active treatment [10,11].

The prevalence of diabetes mellitus continues to increase due to advances in medical technology, longer life expectancy, and changes in diet and lifestyle. As a result, the rate of diabetic nephropathy has steadily increased and it is currently the most common cause of end-stage renal failure worldwide [12,13]. Microalbuminuria is the most important indication of diabetes-related kidney complications, as it appears in the early phase and predicts the progression of complications [2,3,4]. Moreover, the risk of cardiovascular complications increases in patients with diabetes with microalbuminuria; therefore, early screening and prevention are important [5]. Hence, The American Diabetes Association has recommended that a microalbuminuria test be performed following diagnosis and annually thereafter [14].

Abnormal glucose homeostasis in diabetic patients results in various complications as it is accompanied by hyperglycemia caused by a lack of insulin secretion, hypertension, and metabolic disorders [15,16]. Diabetes can induce macrovascular complications that affect the brain, heart, and peripheral vessels and microvascular complications that damage the eyes, kidneys, and nerves [17,18]. Diabetic nephropathy is the most common diabetes-related complication of the microvasculature—it accounts for 30–40% of chronic kidney disease (CKD) cases as the main cause and 45% of end-stage renal disease (ESRD) cases [19]. The prevalence of ESRD in diabetic patients is 10 times higher than that in non-diabetic patients [20]; over the past decade, the incidence of ESRD has rapidly increased as a result of the high incidence of diabetes [20]. Diabetic nephropathy is a severe complication detected in patients with diabetes; it is associated with mortality and increased risks of cardiovascular diseases and ESRD. Hence, renal replacement therapy such as dialysis or transplantation is required [21]. This leads to social burdens and enormous economic costs. Therefore, the risk factors of microalbuminuria should be identified at the early stages of diabetes to prevent the occurrence of complications [22,23].

Related studies have reported that microalbuminuria is an integrated index for renal and cardiovascular risk reduction in patients with type 2 diabetes (T2DM) [24], and that hypertension in patients with diabetes accelerates the onset of diabetic nephropathy in the presence of microalbuminuria [25,26]. Microalbuminuria in patients with diabetes is associated with, among others, diabetes duration, blood pressure, fasting blood sugar (FBS) level, glycosylated hemoglobin (HbA1c) level, serum insulin concentration, dyslipidemia, smoking, and body mass index (BMI) [27,28,29,30,31,32].

However, most studies among patients with diabetes have been conducted in a clinical setting. This study aims to discover appropriate management measures to decelerate the occurrence of complications in consideration of the general characteristics of community-dwelling patients with diabetes. Therefore, we conducted this study to reduce the socioeconomic burden caused by diabetes complications and contribute to public health by identifying the risk factors related to microalbuminuria in patients with T2DM living in the community.

## 2. Materials and Methods

### 2.1. Data Collection and Study Population

The study included adults aged ≥30 years who participated in the eighth Korea National Health and Nutrition Examination Survey (KNHANES) in 2019–2020 [33]. As a nationwide cross-sectional study conducted by the Korea Centers for Disease Control and Prevention based on Article 16 of the National Health Promotion Act, the KNHANES provides reliable statistics that can be used to assess the health and nutritional status of the Korean population [34]. The KNHANES data are useful in the development of health policies that reflect the current health status of people in South Korea. In accordance with the Korean Bioethics and Safety Act, the KNHANES is a government-run research project for public welfare and has been conducted with Institutional Review Board exemption since 2015. The requirement for informed consent was also waived.

Among the participants of the 2019–2020 KNHANES, 11,093 adults aged ≥30 years were selected for this study; among them, 1737 patients diagnosed with T2DM by a physician, receiving hypoglycemic drugs, or with an FBS level of ≥126 mg/dL or HbA1c level of ≥6.5% were selected. In contrast, patients diagnosed with T1DM (including those receiving insulin injection monotherapy), renal disease (CKD or ESRD), cardiovascular disease, or hypertension prior to the diagnosis of diabetes, which could thus affect the level of microalbuminuria, were excluded. Overall, only 539 patients were included in the subsequent analyses.

### 2.2. Assessment of Microalbuminuria Using the ACR Index

In microalbuminuria, the amount of albumin excreted in the urine is 30–300 mg (or 30–300 μg/mg creatinine) per 24 h [1]. Microalbuminuria cannot be detected using the standard urine dipstick test. Microalbuminuria was determined based on the levels of albumin and creatinine in urine using a turbidimetric assay. Therefore, the albumin-to-creatinine ratio (ACR), which is estimated using a random urine sample, is used to screen for microalbuminuria. In this study, a spot urine sample was used to estimate the ACR.

### 2.3. Anthropometric and Biochemical Data

For the blood pressure data, the final systolic blood pressure (SBP) and diastolic blood pressure (DBP) measurements were used. The blood pressure was measured using a manometer (Baumanometer Wall Unit 33, Baum, Methuen, MA, USA) with the arm elevated above the level of the heart after a 5 min rest. To obtain the anthropometric data to estimate the BMI, standard devices and measurement methods were used; the height and weight were measured to the nearest 0.1 cm and 0.1 kg, respectively, using portable measurement equipment (Seca 225, Seca Deutschland, Hamburg, Germany; GL-6000-20, G-Tech, Uijeongbu, Republic of Korea). Obesity was determined based on the BMI, which was calculated by dividing the weight in kilograms by height in meter squared. The BMI was categorized based on the World Health Organization criteria for the Asia-Pacific region. A BMI of <18.5 kg/m^2^ is categorized as underweight, a BMI of 18.5≥–<23 kg/m^2^ is categorized as normal, a BMI of ≥23 kg/m^2^ but <25 kg/m^2^ is categorized as overweight, and a BMI of ≥25 kg/m^2^ is categorized as obese. The waist circumference (WC) cutoff points for Korean individuals were determined according to the criteria suggested by the Korean Society for the Study of Obesity: 90 cm for men and 85 cm for women.

For blood testing, blood samples were collected in the morning after fasting for at least 8 h. The Hitachi Automatic Analyzer 7600-210 (Hitachi, Japan) was used to obtain the measurements. The following biochemical data were collected: FBS, HbA1c, hemoglobin (Hb), serum lipid (triglycerides (TG), total cholesterol (TC), high-density lipoprotein cholesterol (HDL-C), and low-density lipoprotein cholesterol (LDL-C)), serum albumin, and creatinine levels.

### 2.4. Demographic Characteristics

The obtained demographic characteristics included age (≤50, 50–59, 60–69, and ≥70 years), marital status (single, married, separated, or divorced), and economic status (low, middle–low, middle–high, or high) which was also divided into quartiles based on the average monthly household income. The household income was divided by the square root of the number of household members, which is the standard method recommended by the Organization for Economic Cooperation and Development. In terms of smoking status, the patients were categorized as never, ex-, and current smokers.

### 2.5. Statistical Analysis

The collected data were statistically analyzed using R software version 4.1.1 (R Foundation, Vienna, Austria) and according to the guidelines of the 2019–2020 KNHANES for complex sample design. All values in the sample data are expressed as mean and standard error, and the level of significance was set at a *p*-value of <0.05. The general characteristics of the study population were compared using Pearson’s chi-square test for categorical variables and the t-test for continuous variables according to the microalbuminuria status. Logistic regression analysis was performed to assess the risk factors of microalbuminuria in the study population. Using a logistic regression model incorporating the significant independent variables based on the chi-square test and t-test results, the odds ratio (OR) and 95% confidence interval (CI) were obtained through the exponentiation of the estimated regression coefficients. In the logistic regression analysis, the model fitness and significance of each variable were tested to determine the significant variables.

## 3. Results

Among the 539 patients with T2DM in this study, 17.63% had microalbuminuria, 3.71% had albuminuria, and 78.66% had normoalbuminuria. The patients’ mean ages were 64.68 years in the microalbuminuria group and 63.76 years in the normoalbuminuria group. The incidence of microalbuminuria was the highest in individuals aged ≥70 years and in the low-income group. The groups with <10 years and >20 years of diabetes duration had the highest proportions of patients. The microalbuminuria and normoalbuminuria groups did not vary significantly in terms of age, sex, economic status, BMI, WC, or smoking status, although they varied significantly in the duration of diabetes and medication for hypertension (Table 1).

Regarding the health-related characteristics, the mean ACR values were 9.94 mg/g creatinine in the normoalbuminuria group and 96.25 mg/g creatinine in the microalbuminuria group. The between-group variation based on microalbuminuria status was significant for HbA1c, FBS, Hb, TG, HDL-C, LDL-C, serum blood urea nitrogen (BUN), serum creatinine levels, and WC, but not for BMI, TC level, or DBP (Table 2).

When logistic regression was performed to identify significant variables, factors associated with the determination of microalbuminuria were unnecessarily included. To compensate, we excluded diabetes duration, BUN, and creatinine from the significant variables. This improved the fit of the model and the significance of each variable.

**Figure 1:**
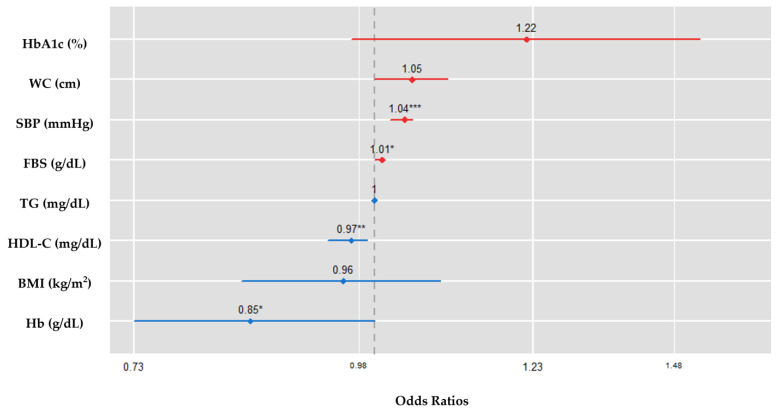
Comparison of the odds ratios by independent variable in the reduced logistic regression model. Abbreviations: HbA1c, glycosylated hemoglobin; WC, waist circumference; SBP, systolic blood pressure; FBS, fasting blood sugar; TG, triglycerides; HDL-C, high density lipoprotein cholesterol; BMI, body mass index; Hb, hemoglobin. * A *p*-value of <0.05 is considered significant. ^**^ A *p*-value of <0.01 is considered significant. ^***^ A *p*-value of <0.001 is considered significant.

Regarding the logistic regression analysis, the ORs were 1.036 (95% CI = 1.019–1.053, *p* < 0.001) for SBP and 0.966 (95% CI = 0.941–0.989, *p* = 0.007), 1.008 (95% CI = 1.002–1.014, *p =* 0.015), and 0.855 (95% CI = 0.729–0.998, *p* = 0.043) for HDL-C, FBS, and Hb levels, respectively (Table 3). For the independent variables in the final model, the OR and 95% CI were visualized in a decreasing order; the results are shown in Figure 1.

## 4. Discussion

Based on the results of this study, the significant risk factors of microalbuminuria in patients with T2DM were SBP and HDL-C, FBS, and Hb levels, in a decreasing order, with the level of significance set at <0.05. The discussion is as follows.

First, as the SBP level increased, the risk of microalbuminuria increased 1.036-fold. Microalbuminuria is an important risk factor that predicts the occurrence of diabetic nephropathy and CKD [2,3,4] and a risk factor of cardiovascular disease associated with hypertension [5]. According to a previous study, the risk of diabetic nephropathy in patients with hypertension and T2DM could be reduced via strict regulation of blood pressure levels [35]; the incidence of microalbuminuria was higher in patients with T2DM with hypertension compared with that in patients with T2DM alone. In the Kidney Disease Improving Global Outcomes guideline [1], the recommended target blood pressure levels to suppress the development of nephropathy and reduce cardiovascular-disease-related mortality are <140/90 mmHg in those with an excretion of <30 mg/g creatinine in the urine and <130/80 mmHg in those with an excretion of ≥30 mg/g creatinine in the urine or a high risk of cardiovascular disease [36]. Thus, for patients with T2DM, blood pressure regulation combined with periodic microalbuminuria tests could be a preventive measure against the development of diabetic nephropathy as well as microalbuminuria.

Second, as the HDL-C level increased, the risk of microalbuminuria decreased 0.996-fold. This finding agrees with the results of a previous study [22] which reported the correlations of microalbuminuria with hypertension, hyperglycemia, a low HDL-C level, and a high TG level, and with the study conducted by Sun et al. [37], which reported a decrease in microalbuminuria caused by a high HDL-C level. A typical patient with T2DM exhibits dyslipidemia with a characteristically low HDL-C level; meanwhile, HDL-C plays a role in the reverse transport of cholesterol, anti-inflammation, and anti-oxidation [38]. The process of reverse cholesterol transport is inhibited by the lack of HDL-C or a related dysfunction, while glomerulosclerosis and tubulointerstitial injury are induced [36]. The reduced anti-oxidation capacity of HDL-C also increases systemic oxidative stress and oxidized LDL levels in the circulation [39]; as the low HDL-C level decreases the glucose absorption in the skeletal muscles and induces the dysfunction of pancreatic β cells, the resulting hyperglycemia and metabolic disorder can damage the glomerular endothelial and tubulointerstitial cells [40]. Through these mechanisms, a low HDL-C level promotes microalbuminuria, hyperglycemia, and diabetic nephropathy [41]. In addition, dyslipidemia is closely associated with an increased risk of diabetes due to the changes in dietary habits and lifestyle; these changes have been correlated with being overweight, insufficient physical activity, smoking, hypertension, and cholesterol levels [39]. For patients with T2DM, increasing the HDL-C level could be a preventive measure against diabetic nephropathy and microalbuminuria.

Third, as the FBS level increased, the risk of microalbuminuria increased 1.008-fold. This finding agrees with the results of a previous study which reported that the risk factors for microalbuminuria were FBS level, blood pressure, old age, TG level, and duration of diabetes [22,23]. Hyperglycemia is a risk factor of the complications of diabetes that play a key role in the onset and development of diabetic nephropathy; hence, patients with T2DM who required strict regulation of blood sugar levels had a significant association with microalbuminuria [42]. Another study supported this association by reporting a correlation between increased excretion of microalbumin and increased levels of blood glucose and insulin [28]. In T2DM, the strict regulation of blood glucose in the early stages and before the onset of diabetic nephropathy prevents the development of diabetic nephropathy [1,14]. Other studies reported that HbA1c, FBS, SBP, and blood lipid levels were risk factors of microalbuminuria [28,29]. In our study, HbA1c was not a significant risk factor for microalbuminuria, but FBS was significant, which means that stricter daily FBS confirmation is required for the long-term glycemic management of HbA1c. The current study showed that the microalbuminuria group had higher FBS levels compared with the normoalbuminuria group (155 vs. 134 mg/dL). This is thought to reflect the importance of FBS in daily glycemic management as a risk factor for microalbuminuria, which implies that hyperglycemia or the inadequate regulation of blood glucose level could cause diabetic nephropathy with microalbuminuria.

As such, the ultimate goals of T2DM treatment are to prevent potential diabetic complications and maintain a healthy life through the regulation of blood glucose, blood pressure, and blood cholesterol levels [43]. To achieve these goals, palliative therapy such as blood glucose and blood pressure control, treatment of dyslipidemia, lifestyle modification, and regulation of dietary sodium intake are critical. Hence, the early detection of microalbuminuria and public health education and management are necessary to prevent diabetic nephropathy. Thus, a mobile health program for the systematic management of patients with T2DM should be developed; moreover, e-learning education and notifications regarding the probability of developing microalbuminuria and related complications should be provided as part of an integrated healthcare service.

Finally, as the Hb level increased, the risk of microalbuminuria decreased 0.885-fold. These results show that the hemoglobin level was associated with low HDL-C and high FBS and SBP levels which is considered to be significant due to vascular damage. According to a previous study into diabetes, a lower hemoglobin level was a risk factor of the progression of diabetic nephropathy [44], and it was increased in the prevalence of anemia in microalbuminuria compared to normoalbuminuria [45]. Anemia was a risk factor for albuminuria and kidney damage in patients with T2DM [46]. Furthermore, albuminuria is a risk factor for anemia in the CKD [47]; this is mainly due to reduced erythropoietin formation in the kidneys. Renal anemia appears early in the CKD process and worsens as it progresses. Given that the signs and symptoms of anemia in diabetes depend on the period in which Hb reduction is advanced and begin slowly, anemia associated with CKD is often asymptomatic and is only detected via routine blood tests. Other studies have shown that Hb, albuminuria, and kidney function are also strongly associated with cardiovascular risk [48]. This is important because delayed diagnosis and treatment of anemia associated with kidney disease may increase the risk of cardiovascular complications. Therefore, when microalbuminuria occurs in patients with T2DM, follow-up with anemia testing may help to prevent diabetes complications.

In addition, we found that the incidence of microalbuminuria was highest in patients aged ≥70 years and was higher in patients with a duration of diabetes of <10 years or >20 years compared with that in patients with a diabetes duration of 10–20 years. These results indicate that patients with a 10–20 year duration of diabetes had a greater interest in undergoing diabetes management as their diabetes had already progressed. In patients with a <10 year duration of diabetes, periodic tests could be inadequate or their diabetic management could have been neglected, thus suggesting a need for the intensive prevention and management of diabetic complications. In particular, it is worth noting that the incidence of microalbuminuria was the highest in patients with T2DM with low income, which coincided with a study on the differences in the incidence of microalbuminuria according to socioeconomic status [49,50]. This finding suggests the need for active public health strategies and support regarding the early detection of microalbuminuria and the prevention of diabetic nephropathy in patients with T2DM with low income.

The strength of this study is that the sample population was selected from the participants of KNHANES, an extensive national survey, which supports the possibility of generalizing the results of the study. Meanwhile, this study has several limitations. The cause–effect relationships across factors involved in microalbuminuria could not be determined as the study was cross-sectional in nature. Second, the classification of microalbuminuria could have been inaccurate as the microalbuminuria test was performed only once. Third, the effects of drugs on diabetes, hypertension, and hyperlipidemia could not be taken into account. Lastly, transient false-positive results were possibly obtained due to the performance of intense physical exercise, the occurrence of fever, or the development of urinary tract infection.

## 5. Conclusions

The risk factors of microalbuminuria in patients with T2DM were SBP and HDL-C, FBS, and Hb levels, the most important finding being that the Hb level was identified as a risk factor. Nonetheless, verification is required through subsequent studies on the association between microalbuminuria and anemia. In addition, the need for public health strategies that consider age and income level in community-dwelling patients with T2DM was confirmed.

Based on the results of this study, the authors would like to make the following suggestions. First, efforts should be made to reduce the inequalities in healthcare for patients with T2DM and low socioeconomic status and the use of healthcare services for the aged population. Second, further studies should develop a digital health program to reduce the risk factors of microalbuminuria in patients with T2DM.

## Figures and Tables

**Table 1:** General characteristics of the participants according to microalbuminuria status.

Variables		Microalbuminuria	Normoalbuminuria	Chi-Square Test *(p*-Value)
		n (%)	n (%)	
Age (years)	<50	9 (9.47%)	48 (11.32%)	2.43 (0.49)
	50~59	17 (17.89%)	87 (20.52%)	
	60~69	28 (29.47%)	142 (33.49%)	
	≥70	41 (43.16%)	147 (34.67%)	
Sex	Male	47 (49.47%)	208 (49.06%)	0.01 (0.94)
	Female	48 (50.53%)	216 (50.94%)	
Economic status	High	20 (21.05%)	106 (25.00%)	3.46 (0.33)
	Middle–High	23 (24.21%)	110 (25.94%)	
	Middle–Low	21 (22.11%)	108 (25.47%)	
	Low	31 (32.63%)	100 (23.58%)	
Duration of diabetes	<10	34 (35.79%)	235 (55.43%)	**18.36 (<0.001)**
	10~20	27 (28.42%)	115 (27.12%)	
	>20	34 (35.79%)	74 (17.45%)	
Smoking status	Current smoker	19 (3.66%)	71 (13.68%)	1.21 (0.54)
	Ex-smoker	23 (4.43%)	124 (23.89%)	
	Never smoker	53 (10.21%)	229 (44.12%)	
Medication for diabetes	Yes No	90 (17.34%) 5 (0.96%)	395 (76.11%) 29 (5.59%)	0.32 (0.57)
Medication for hypertension	Yes No	27 (5.20%) 68 (13.10%)	68 (13.10%) 356 (68.59%)	**7.96 (0.00)**

Bold: *p*-values < 0.05 were considered statistically significant.

**Table 2:** Clinical characteristics of the participants according to the microalbuminuria status.

Variables	Microalbuminuria	Normoalbuminuria	*t*-Test *(p*-Value)
	M ± SD	M ± SD	
Glycosylated hemoglobin (%)	7.78 ± 1.31	7.20 ± 1.20	**13.95 (<0.001)**
Fasting blood sugar (g/dL)	155.60 ± 58.32	134.96 ± 38.45	**9.46 (<0.001)**
Hemoglobin (g/dL)	13.52 ± 1.59	13.75 ± 1.59	**7.98 (<0.001)**
Body mass index (kg/m^2^)	24.99 ± 3.09	24.42 ± 3.23	2.31 (0.10)
Triglycerides (mg/dL)	145.75 ± 91.75	142.49 ± 111.84	**8.72 (<0.001)**
Total cholesterol (mg/dL)	165.86 ± 45.00	165.34 ± 37.64	1.19 (0.31)
HDL-C (mg/dL)	44.25 ± 10.53	47.89 ± 11.16	**5.08 (<0.001)**
LDL-C (mg/dL)	92.46 ± 39.04	88.96 ± 34.01	0.60 (0.55)
Blood urea nitrogen (mg/dL)	17.29 ± 6.08	16.96 ± 5.24	**6.67 (<0.001)**
Serum creatinine (mg/dL)	0.86 ± 0.32	0.81 ± 0.21	**7.85 (<0.001)**
Waist circumference (cm)	90.20 ± 8.15	87.83 ± 8.87	**5.99 (<0.001)**
Systolic blood pressure (mmHg)	128.42 ± 16.56	120.91 ± 14.16	**11.21 (<0.001)**
Diastolic blood pressure (mmHg)	71.69 ± 9.79	72.02 ± 9.68	0.07 (0.93)

Abbreviations: M ± SD, mean ± standard deviation; HDL-C, high-density lipoprotein cholesterol; LDL-C, low-density lipoprotein cholesterol; bold: *p*-values < 0.05 were considered statistically significant.

**Table 3:** Odds ratios for the occurrence of microalbuminuria.

Variable	Unadjusted	Adjusted	*p*-Value
	OR (95% CI)	OR (95% CI)	
Glycosylated hemoglobin (%)	1.139 (0.851, 1.51)	1.216 (0.965, 1.524)	0.092
Fasting blood sugar (g/dL)	1.010 (1.005, 1.015)	1.008 (1.002, 1.014)	**0.015**
Hemoglobin (g/dL)	0.885 (0.749, 1.043)	0.855 (0.729, 0.998)	**0.043**
Body mass index (kg/m^2^)	0.971 (0.996, 1.001)	0.956 (0.84, 1.085)	0.489
Triglycerides (mg/dL)	0.979 (0.929, 1.031)	0.999 (0.996, 1.001)	0.303
HDL-C (mg/dL)	0.973 (0.949, 0.995)	0.966 (0.941, 0.989)	**0.007**
Waist circumference (cm)	0.978 (0.951, 1.005)	1.045 (0.995, 1.098)	0.078
Systolic blood pressure (mmHg)	1.032 (1.015, 1.060)	1.036 (1.019, 1.053)	**<0.001**

Abbreviations: OR, odds ratio; CI, confidence interval; HDL-C, high-density lipoprotein cholesterol. Bold: *p*-value of <0.05 is considered significant.

## Data Availability

Not applicable.
